# Rapid screening of photoactivatable metallodrugs: photonic crystal fibre microflow reactor coupled to ESI mass spectrometry†
†Electronic supplementary information (ESI) available. See DOI: 10.1039/c7ra06735f


**DOI:** 10.1039/c7ra06735f

**Published:** 2017-07-26

**Authors:** Ruth J. McQuitty, Sarah Unterkofler, Tijmen G. Euser, Philip St.J. Russell, Peter J. Sadler

**Affiliations:** a Department of Chemistry, University of Warwick, Gibbet Hill Road, Coventry CV7 4AL, UK. Email: P.J.Sadler@warwick.ac.uk; b Max-Planck Institute for the Science of Light, Staudtstrasse 2, D-91058 Erlangen, Germany. Email: Philip.Russell@mpl.mpg.de; c NanoPhotonics Group, Cavendish Laboratory, J J Thomson Avenue, Cambridge CB3 0HE, UK

## Abstract

We explore the efficacy of a hyphenated photonic crystal fibre microflow reactor – high-resolution mass spectrometer system as a method for screening the activity of potential new photoactivatable drugs. The use of light to activate drugs is an area of current development as it offers the possibility of reduced side effects due to improved spatial and temporal targeting and novel mechanisms of anticancer activity. The di-nuclear ruthenium complex [{(η^6^-indan)RuCl}_2_(μ-2,3-dpp)](PF_6_)_2_, previously studied by Magennis *et al.* (*Inorg. Chem.*, 2007, **46**, 5059) is used as a model drug to compare the system to standard irradiation techniques. The photodecomposition pathways using blue light radiation are the same for PCF and conventional cuvette methods. Reactions in the presence of small biomolecules 5′-guanosine monophosphate (5′-GMP), 5′-adenosine monophosphate (5′-AMP), l-cysteine (l-Cys) and glutathione (γ-l-glutamyl-l-cysteinyl-glycine, GSH) were studied. The complex was found to bind to nucleobases in the dark and this binding increased upon irradiation with 488 nm light, forming the adducts [(η^6^-indan)Ru_2_(μ-2,3-dpp) + 5′-GMP]^2+^ and [(η^6^-indan)Ru + (5′-AMP)]^+^. These findings are consistent with studies using conventional methods. The dinuclear complex also binds strongly to GSH after irradiation, a possible explanation for its lack of potency in cell line testing. The use of the PCF-MS system dramatically reduced the sample volume required and reduced the irradiation time by four orders of magnitude from 14 hours to 12 seconds. However, the reduced sample volume also results in a reduced MS signal intensity. The dead time of the combined system is 15 min, limited by the intrinsic dead volume of the HR-MS.

## Abstract 2

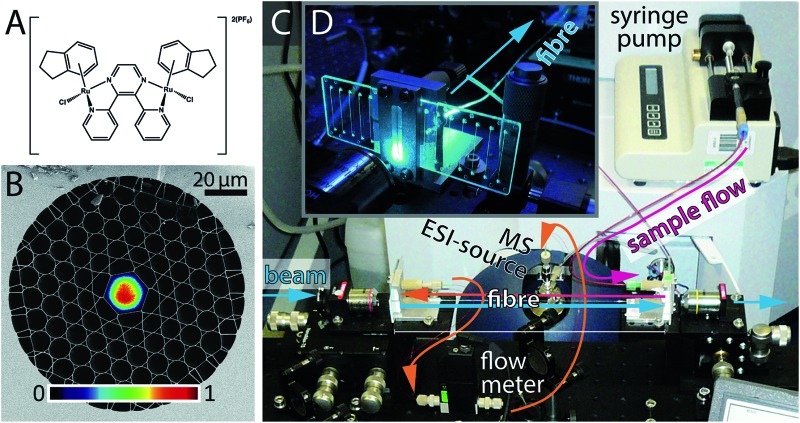
We explore the efficacy of a hyphenated photonic crystal fibre microflow reactor – high-resolution mass spectrometer system as a method for screening the activity of potential new photoactivatable drugs.

## Introduction

The use of light activated drugs in the form of photodynamic therapy (PDT) is well established in the clinic, having gained FDA approval in the US in 1995.^1^ Photodynamic therapy involves the use of a photosensitiser that is irradiated with light, normally within the ‘phototherapeutic window’ of 650–850 nm. The excited photosensitiser then generates reactive oxygen species, especially ^1^O_2_, which cause irreparable damage to the tumour cell and induce cell death.^2^ Although a proven treatment, PDT does have its limitations, such as photosensitivity of the patient after treatment and the reliance of the treatment on the presence of oxygen, which means that its efficacy is curbed in the hypoxic regions of solid tumours.^3^

The search for improved photoactive drugs with a wider spectrum of activity and that possess a mechanism of action independent of the presence of oxygen has led to many new photoactive agents being synthesised. Some of these make use of the unique photochemistry of inorganic compounds, utilising a range of metal centres such as iridium,^4^ ruthenium,^5^ platinum,^6^ and dinuclear mixed-metal complexes.^7^–^10^ Examples from our own work include photoactivatable diazido Pt(iv) complexes,^11^ and organometallic Ru(ii) compounds.^12^,^13^ This work has resulted in promising drug candidates, such as *trans*,*trans*,*trans*-[Pt(py)_2_(N_3_)_2_(OH)_2_] which is active at micromolar concentrations against a range of cancer cell lines when irradiated with low doses of blue light.^14^

Since the chemistry of electronically-excited states of photoactive metal complexes differs from that of the ground state, photoproducts are likely to be produced which can affect cellular biochemical pathways in novel ways.^15^ Such behaviour may be advantageous for avoiding cross-resistance with existing drugs. For example, combatting resistance to current platinum drugs is an important clinical problem.^16^ Hence there is a need to devise methods for analysis of the photodecomposition pathways of photochemotherapeutic agents on a wide range of timescales. Conventional methods often involve the separate irradiation and subsequent analysis of a photoactive compound in solution, require relatively high sample volumes (at least 600 μL), and can involve lengthy sample handling procedures. The aim of the current work is to utilise the unique properties of liquid-filled hollow-core photonic crystal fibres (HC-PCFs) to act as micro flow-reactors for the efficient activation and analysis of photoactivatable drugs on a much shorter timescale than is possible with conventional methods.

**Fig. 1:**
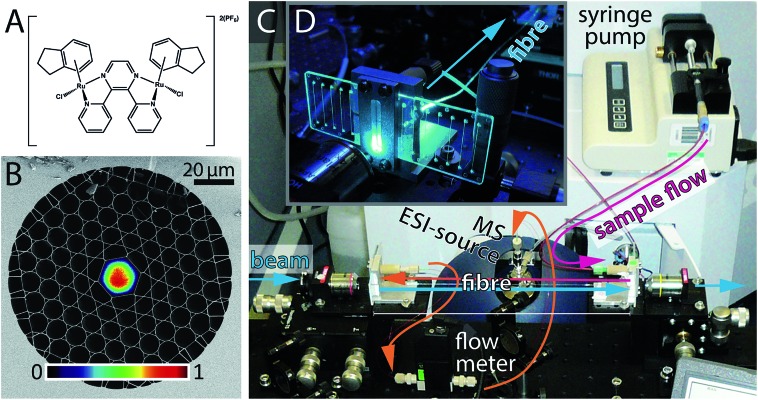
(A) Structure of **1**, [{(η^6^-indan)RuCl}_2_(μ-2,3-dpp)](PF_6_)_2_. (B) Scanning electron micrograph of the kagomé-lattice HC-PCF. Grey areas correspond to fused silica glass and black areas correspond to holes, which in our experiment are filled with sample solution. The overlayed image is the optical mode at 488 nm wavelength, measured by a CCD camera. (C) Photograph of the experimental setup. Custom-made optofluidic interface mounts (see D) are used to couple light into the optofluidic PCF flow reactor. (D) Photograph of one optofluidic interface mount, accommodating an off-the-shelf straight-channel optofluidic chip.

HC-PCFs are fibres constructed from silica, comprising a ∼20 μm-sized hollow core channel, surrounded by a regular two-dimensional arrangement of smaller channels (total diameter *ca.* 200 μm),^17^ see Fig. 1B. The HC-PCF design, such as the core size, the cladding pitch, and the thickness of the glass webs surrounding the hollow core, can be adjusted such that a desired range of wavelengths is guided along the core with negligible losses.^18^–^20^ The same guiding mechanism, though over a shifted wavelength range, applies when the microstructure is filled with a liquid,^21^,^22^ meaning that light can propagate in well-defined optical modes along a microscale microfluidic channel, as shown in Fig. 1B.

Such optofluidic HC-PCFs are outstanding photochemical microreactors, that outperform on-chip optofluidic waveguides^23^,^24^ in several ways.^25^,^26^ First of all, the reaction volume per cm interaction length is reduced to a few nL cm^–1^ enabling studies on minute sample volumes. In terms of photochemistry, the available optical power *P* is confined to the small cross-sectional area of the core (*A*_core_ ∼ 300 × 10^–12^ m^2^), resulting in a five orders of magnitude larger average irradiance (*I* = *P*/*A*_core_), as compared to a standard size cuvette irradiated by a free-space beam. As a result, much lower laser powers are required to achieve the same excitation conditions. Finally, HC-PCF allows long optical path lengths that can be exploited for enhanced detection sensitivity. Additional advantages are that HC-PCFs are easily fabricated by established procedures^27^ from high-quality fused silica glass, featuring negligible scattering and absorbance in the UV-Vis range, as well as chemical inertness.

Recent experimental studies have proven the efficacy of optofluidic PCFs as efficient photochemical reactors. Using simultaneous in-fibre absorption spectroscopy on a fixed, static sample volume, efficient photoconversion of low quantum yield reactions,^25^ photo-induced homogeneous catalysis,^28^ and monitoring of the reaction kinetics of photoswitchable dyes with sub-picomole sensitivity have been successfully demonstrated.^29^ Cubillas *et al.* have published a comprehensive review of the uses of PCFs for photochemistry and sensing applications.^30^

The analysis of photodecomposition pathways in HC-PCFs usually relies on absorption spectroscopy within the fibre core. While providing some useful *in situ* information, an important limitation is that it is very difficult to deduce structural information of reaction products from the absorption spectrum alone. For this reason, we have recently combined a continuous-flow HC-PCF microreactor with non-optical analysis methods to characterise unknown species more fully. A piece of optofluidic HC-PCF was interfaced with a low-volume microfluidic circuit, enabling photochemical reaction products to be fed directly into a mass spectrometer (MS),^31^ see Fig. 1D. As a proof-of-principle, the well-known photoaquation reaction of cyanocobalamin (vitamin B_12_) to aquacobalamin was studied and found to be in excellent agreement with conventional irradiation methods.^32^ Importantly, the amount of sample required was reduced 50 times when compared to the conventional batch procedure using a cuvette.

In this study, we apply this novel optofluidic system to gain new insight into the properties of potential photoactivatable drugs. The ruthenium complex **1**, shown in Fig. 1A, is a dinuclear complex previously studied by Magennis *et al.*^33^ Upon irradiation, **1** was found to lose an indane ligand and bind to DNA. The complex can also undergo aquation in aqueous solution in the dark, resulting in the loss of the chlorido ligands. We have studied he photoactivation of **1** in the presence of a range of biomolecules as a guide to its possible intracellular behaviour.

## Experimental section

### Materials

All water used was doubly deionised water (DDW), purified using a Multipore Milli Q and a USF Elga UHQ water deionisers. Microfluidic mounts were constructed in-house at the Max Planck Institute for the Science of Light in Erlangen. The microfluidic chips were purchased from microfluidic ChipShop. For sample introduction, a 500 μL-glass syringe from Hamilton was used and a syringe pump from kdScientific. Sodium salt 5′-GMP (99.99%) and l-cysteine (99.99%) were purchased from Sigma Aldrich. Both the 5′-AMP (99.99%) and glutathione (GSH, 99.99%) were purchased from Acros Organics. Complex **1** ([{(η^6^-indan)RuCl}_2_(μ-2,3-dpp)](PF_6_)_2_) was synthesised according to the literature method.^33^

### Sample preparation

All MS samples were filtered using NALGENE 0.2 μm PES filters and then centrifuged at 14 000 rpm, 5 °C for 10 min. The pH of samples was measured using a Mettler-Toledo glass microelectrode connected to a Martini instruments Mi150 pH/temperature bench meter calibrated at pH 4, 7 and 10 using pH buffers from Mettler-Toledo.

### Mass spectrometry

All mass spectra were obtained on a Bruker MaXis high-resolution mass spectrometer (HR-MS). Samples were introduced to the MS at a flow rate of 100 μL h^–1^. The acquisition parameters for spectra obtained in the positive mode were as follows: scan range 50–3000 *m*/*z*, set capillary 3000 V, end plate off-set –500 V, nebuliser pressure 0.4 bar, dry heater 180 °C, and dry gas 4.0 L min^–1^. All spectra were processed using Bruker Daltonics Analysis software and Origin Pro 8.1.

### Optofluidic setup

A photograph of the optofluidic photochemical microflow reactor is shown in Fig. 1C and D. Details of the implementation, especially of the optofluidic interface between the optical fibre facets and the microfluidic circuitry (PEEK tubing equipment by VICI and Upchurch Scientific) *via* off-the-shelf microfluidic chips (microfluidic ChipShop), can be found in Unterkofler *et al.*^31^ The HC-PCF used in the experiments was a *L* ≈ 15 cm long kagomé-type HC-PCF^20^ with a core diameter of 19.7 μm (Fig. 1A). This corresponds to a fibre-dead volume of ∼1.4 μL in the cladding holes and ∼50 nL in the optofluidic core. The fibre guides blue light (488 nm) in a well-defined optical mode (see Fig. 1B) with waveguide loss of 3.5 dB m^–1^, meaning that the transmission losses through the 15 cm long fibre are below 12%. The inner diameter (100 μm) and length (60 cm) of the PEEK tubing connecting the microfluidic chip with the MS, were kept short to minimise the dead volume of the set-up. In this way, the dead volume of the circuitry between fibre exit and mass spectrometer was kept below 10 μL. A continuous flow rate of *φ*_tot_ = 100 μL h^–1^ = 27.7 nL s^–1^, was established using a syringe pump. The flow rate was continuously monitored with an inline microflow meter (Sensirion model SLG1430-150) placed just before the mass spectrometer. The total sample volume flow through the fibre consists of a portion flowing through the illuminated region in the core, *φ*_core_, and of a portion flowing through the dark cladding holes, *φ*_cladd_. By application of Hagen–Poiseuille's law for laminar flow in a parallel circuit of tubes we find *φ*_cladd_ = 5.6*φ*_core_ for the fibre used. This gave rise to a flow velocity of 1.25 cm s^–1^ through the fibre core and hence to a sample transit time through the core *τ*_trans_ of ∼12 seconds. This, combined with the dead volume of the rest of the system gives and overall dead time of ∼6 min. However, the time between sample introduction and observation of a stable mass spectrum was found to be in the order of 15 min. This is likely due to the intrinsic dead volume of the HR-MS itself and the time needed for the spray to stabilise.

### Irradiation

The photochemical conversion rate depends both on external factors as well as on intrinsic parameters relating to the molecule itself. Molecule-intrinsic parameters are characterised by the molar extinction coefficient *ε*, and the quantum yield *Φ*, *i.e.* the fraction of absorbed photons that lead to the desired reaction. At an excitation wavelength of 488 nm, *ε* = 1880 M^–1^ cm^–1^ and *Φ* = 10^–4^ for complex **1**.^33^ External factors are the excitation wavelength *λ* and the laser power *P*, which is directly proportional to the irradiance *I via I* = *P*/*A*_core_. The light source used here was a blue 488 nm laser from Toptica photonics. Prior to each individual experiment, the laser power was adjusted such that full photochemical conversion was achieved in the core, 1 – *C*(*τ*_trans_)/*C*_0_ → 1 (for details see ref. 26). This model takes also into account the length of the fibre and the fraction of light launched into the fibre core, which varied from 50 to 75% between experimental runs. Required powers *P*_0_ were calculated to be between ∼8–10 mW for the experiments discussed below, leading to irradiances *I*_0_ on the order of 2400–3000 W cm^–2^, much larger than can be achieved in conventional cuvette-based methods.

## Results and discussion

The photoactive dinuclear organometallic complex **1**, [{(η^6^-indan)RuCl}_2_(μ-2,3-dpp)](PF_6_)_2_ (Fig. 1A), was selected for study by PCF-coupled-MS. The photoactivation, aquation and plasmid DNA binding properties of this compound have been reported,^33^ and provide a basis for the reactions investigated here. The aim of this series of experiments was to use the PCF as a microreactor to photoactivate **1** in the presence of a range of small biomolecules that are relevant to possible reactions in cells.

Upon irradiation, **1** loses an indane ligand and can bind to DNA.^33^ The complex can also undergo aquation in aqueous solution in the dark. Aquation can be prevented by the presence of a high concentration of Cl^–^ ions. However, this approach is impractical, as the mass spectrometer does not tolerate high levels of salt. There were controls using conventional methods for each experiment and dark controls using both techniques. Since data from both the PCF and the conventional methods were acquired over time from aqueous solutions, account has to be taken of the possible aquation of **1** in both dark and irradiated samples.

**Fig. 2:**
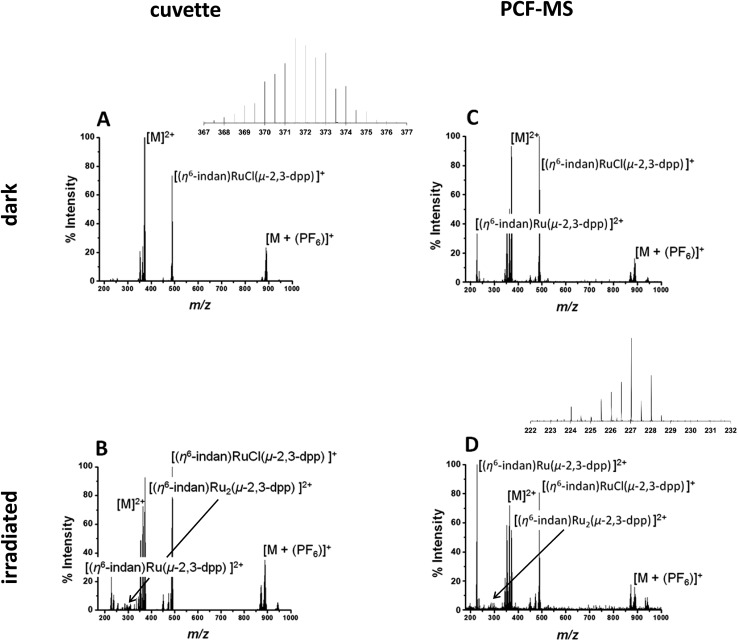
(A and B) Direct infusion of solutions of **1**, 250 μM in DDW. Peak assignments in spectrum (A) of the solution in the dark 372.0 *m*/*z* [M]^2+^ (with inset of isotopic pattern), 888.96 *m*/*z* [M + (PF_6_)]^+^, and at 489.01 *m*/*z* fragment ion [(η^6^-indan)RuCl(μ-2,3-dpp)]^+^. Spectrum (B) of **1** 250 μM in DDW irradiated at 488 nm light (5 mW) for 14 h, in a cuvette showing the appearance of a peaks at 227.04 *m*/*z* assigned to [(η^6^-indan)Ru(2,3-dpp)]^2+^, 276.99 *m*/*z* assignable to [(η^6^-indan)Ru_2_(μ-2,3-dpp)]^2+^. (C and D) PCF-MS system mass spectra of 250 μM **1** in DDW: (C) solution in the dark; peaks assigned 372.0 *m*/*z* as [M]^2+^, 888.96 *m*/*z* as [M + (PF_6_)]^+^ and fragment ion at 489.01 *m*/*z* ([(η^6^-indan)RuCl(μ-2,3-dpp)]^+^); (D) after 12 s of irradiation at 488 nm in the HC-PCF flow reactor, the new peaks at 227.04 *m*/*z* assigned to [(η^6^-indan)Ru(2,3-dpp)]^2+^ (with inset of isotopic pattern) and at 276.99 *m*/*z* is assigned to [(η^6^-indan)Ru_2_(μ-2,3-dpp)]^2+^.

The direct infusion (non-irradiated) controls, Fig. 2A, indicate that the fragmentation process for this molecule is different from its photodissociation and aquation pathways. Complex **1** and its products have complicated isotopic patterns; therefore all of the *m*/*z* values reported refer to the mono-isotopic peak for clarity. The main ions detected in the mass spectrum from dark solutions are the doubly-charged molecular ion at 372.0 *m*/*z* ([M]^2+^), a singly-charged PF_6_ adduct at 888.96 *m*/*z* ([M + (PF_6_)]^+^) and a singly-charged fragment ion at 489.01 *m*/*z* ([(η^6^-indan)RuCl(μ-2,3-dpp)]^+^). There are also two sets of peaks with slightly lower *m*/*z* than the molecular ion at 363.51 *m*/*z* and 353.00 *m*/*z* that can be attributed to aquation of **1** and show the loss of at least one Cl^–^ ion. In summary, the mass spectra of dark solutions of **1** show evidence for aquation and the loss of chloride ligands and fragmentation into mononuclear species. However, no loss of an indane ligand from the ruthenium was detected (Fig. 2A).

A 250 μM solution of **1** in DDW was irradiated with 488 nm light (5 mW) for 14 h in a cuvette and the resulting spectrum is shown in Fig. 2B. The intensities of the peaks assignable to aquated **1** are higher in the spectrum of the irradiated solution that for the complex in the dark. Peaks for the species at 227.04 *m*/*z* assigned to the mononuclear complex [(η^6^-indan)Ru(2,3-dpp)]^2+^ are more intense. The major difference in the spectra is the peak at 276.99 *m*/*z* that is observed after irradiation and is assigned as [(η^6^-indan)Ru_2_(μ-2,3-dpp)]^2+^. This is significant as it shows the loss of an indane ligand from **1** and provides a marker for the photodecomposition of the complex, since arene loss is not observed under dark conditions. Suggested structures for these fragments are shown in Table S1 in the ESI.†

The experiment was then repeated in the integrated PCF-MS system. The dark spectrum in Fig. 2C shows that the only difference from the control experiment in Fig. 2A is an increase of species resulting from aquation at 363.51 *m*/*z*. This is to be expected as the compound in Fig. 2A had been in solution for a longer period of time as it was irradiated for longer. There is also a drop in overall intensities of peaks for the PCF-spectrum, which is to be expected when a reaction is transferred to the integrated system, as well as loss of intensity due to hydrolysis of the initial species. The intensity of the species [(η^6^-indan)Ru(2,3-dpp)]^2+^ (227.04 *m*/*z*) is higher for the sample injected in the dark in the PCF experiment than when directly infused in the dark. The mass spectrum of the irradiated PCF sample in Fig. 2D displays the same species as the irradiated control in Fig. 2B, including a significant increase in the peak at 227.04 *m*/*z*, illustrating that the PCF system does not affect the course of the reaction taking place.

**Fig. 3:**
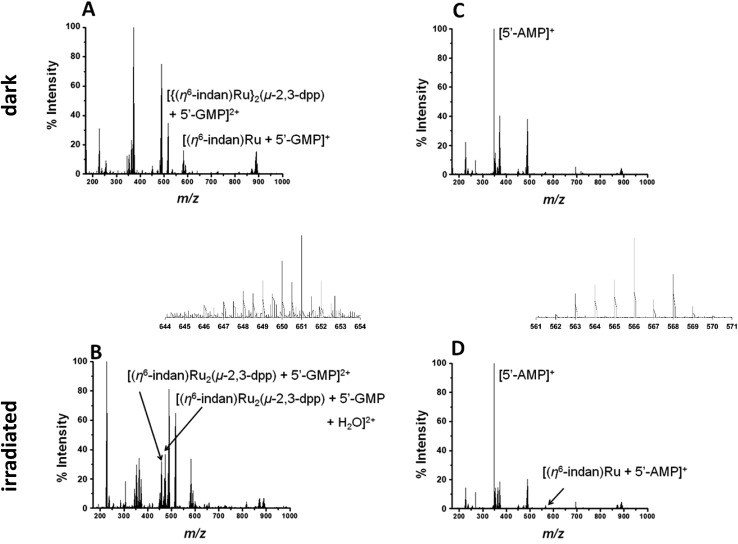
(A and B) PCF-MS of 250 μM **1** and 500 μM 5′-GMP in DDW. Spectrum (A) dark solution peak at 517.6 *m*/*z* assigned as [{(η^6^-indan)Ru}_2_(μ-2,3-dpp) + 5′-GMP]^2+^, the species at 582.03 *m*/*z* is [(η^6^-indan)Ru + 5′-GMP]^+^, and spectrum (B) is after 12 s of irradiation at 488 nm in the HC-PCF flow reactor, new peaks 640.04 *m*/*z* and 649.04 *m*/*z* (shown in inset) are assigned as [(η^6^-indan)Ru_2_(μ-2,3-dpp) + 2(5′-GMP)]^2+^ and [(η^6^-indan)Ru_2_(μ-2,3-dpp) + 2(5′-GMP) + H_2_O]^2+^, respectively. (C and D) PCF-MS of 250 μM **1** and 500 μM 5′-AMP in DDW. Spectrum (C) dark solution, a peak is detected at 348.07 *m*/*z* ([5′-AMP + H]^+^), and spectrum (D) is after 12 s of irradiation at 488 nm in the HC-PCF flow reactor, the new peak at 566.04 *m*/*z* (shown in inset) is assigned as [(η^6^-indan)Ru + (5′-AMP)]^+^.

Metal-based anticancer drugs that are currently in the clinic are known to bind to DNA.^34^–^36^ The DNA binding properties of **1** were modelled by irradiating it in the presence of two nucleobases with the most electron-dense donor sites (5′-GMP and 5′-AMP). Initially two molar equivalents of 5′-GMP were added to an aqueous solution of **1** (500 μM nucleobase and 250 μM of complex). Spectra in the dark were obtained for both PCF-MS and conventional experiments. The samples were irradiated as follows: 14 h at 5 mW in a cuvette for the conventional measurement and 12 s during transfer through the PCF microreactor. The resulting data are summarized in Fig. 3. Once again there was close agreement between the conventional method and the PCF-MS experiment, the only differences being a drop in peak intensity for the PCF experiments and the appearance of the species at 658.6 *m*/*z* in only the spectrum from the PCF irradiated sample.

In the dark spectrum in Fig. 3A, there is the appearance of a new peak at 517.6 *m*/*z* [{(η^6^-indan)Ru}_2_(μ-2,3-dpp) + 5′-GMP]^2+^. This suggests that 5′-GMP binds to **1** in the dark, probably after aquation. This species appears to be fragmented in the mass spectrometer, giving rise to the ion at 582.03 *m*/*z*. Upon irradiation by conventional and PCF-MS methods new peaks were observed at 458.51 *m*/*z* and 467.5 2 *m*/*z* that are assigned as [(η^6^-indan)Ru_2_(μ-2,3-dpp) + 5′-GMP]^2+^ and [(η^6^-indan)Ru_2_(μ-2,3-dpp) + 5′-GMP + H_2_O]^2+^ (see Fig. 3B). These species are indicative of post-irradiation binding due to the loss of an indan ring. This conclusion is further supported by the presence of a small peak at 640.04 *m*/*z*, corresponding to a doubly-charged species assigned as [(η^6^-indan)Ru_2_(μ-2,3-dpp) + 2(5′-GMP)]^2+^ and at 649.04 *m*/*z*, assigned as [(η^6^-indan)Ru_2_(μ-2,3-dpp) + 2(5′-GMP) + H_2_O]^2+^. The species at 658.6 *m*/*z* is present only in the PCF mass spectrum and is of relatively low intensity; this is assigned as [(η^6^-indan)Ru_2_(μ-2,3-dpp) + 2(5′-GMP) + H_2_O + 2H^+^]^2+^. The binding of 5′-GMP that occurs after aquation of the complex also increases in the post-irradiation spectra.

Complex **1** was then irradiated in the presence of 5′-AMP under the same conditions as those for 5′-GMP (250 μM **1** and 500 μM nucleobase). In the dark there appeared to be no reaction between **1** and 5′-AMP, see Fig. 3C. After irradiation by both methods, a species appeared at 623.55 *m*/*z*; this is assigned as [(η^6^-indan)Ru_2_(μ-2,3-dpp) + 2(5′-AMP)]^2+^ (Fig. 3B and D). The low intensity of this peak in the PCF-MS spectrum and low signal-to-noise ratio did not allow this peak to be assigned based on these data alone. However, the assignment was confirmed using the sample irradiated by conventional methods. This was also the case for the peak at 566.04 *m*/*z*, assigned as [(η^6^-indan)Ru + (5′-AMP)]^+^.

**Fig. 4:**
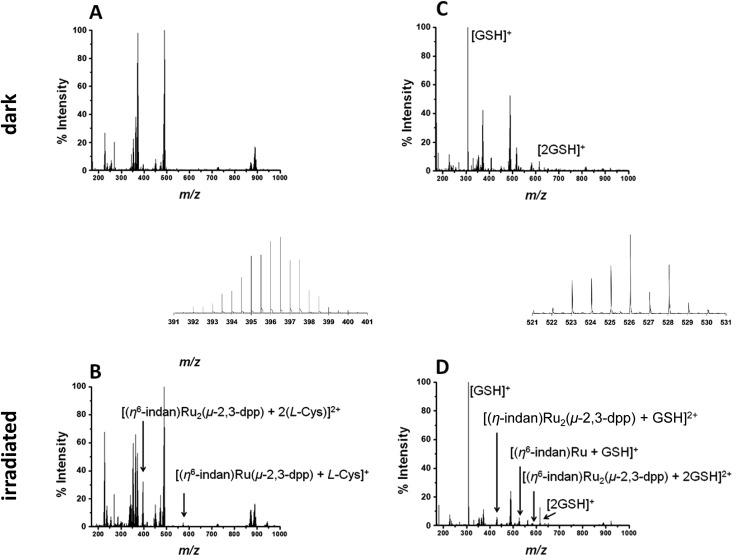
(A and B) PCF-MS for the reaction of 250 μM **1** and 500 μM l-Cys in DDW. Spectrum (A) is for the solution in the dark and spectrum (B) is after 15 min irradiation with 488 nm light (5 mW) in a cuvette. The new peaks in spectrum (B) are assigned as 396.52 *m*/*z* (shown in inset), [(η^6^-indan)Ru_2_(μ-2,3-dpp) + 2(l-Cys)]^2+^ and 574.09 *m*/*z*, [(η^6^-indan)Ru(μ-2,3-dpp) + (l-Cys)]^+^. (C and D) PCF-MS of 250 μM **1** and 500 μM GSH in DDW. Spectrum (C) is from the solution in the dark, with peaks 208.09 *m*/*z* assigned as [GSH + H]^+^, 615.17 *m*/*z* as [2GSH + H]^+^ and spectrum (D) is after 12 s of irradiation at 488 nm in the HC-PCF flow reactor, peaks at 430.52 *m*/*z*, 526.06 *m*/*z* (shown in inset) and 584.06 *m*/*z* are assigned to [(η^6^-indan)Ru_2_(μ-2,3-dpp) + GSH]^2+^, [(η^6^-indan)Ru + GSH]^+^ and [(η^6^-indan)Ru_2_(μ-2,3-dpp) + 2GSH]^2+^, respectively.

To study the interaction with amino acids, complex **1** was irradiated in the presence of l-cysteine at a molar ratio of 2 : 1 (500 μM amino acid, 250 μM complex). The PCF-MS sample was incubated at ambient temperature for 30 min in the dark, and 14 h for the conventional measurement. There appeared to be no reaction in the dark between **1** and l-cysteine, see Fig. 4A.

Upon irradiation by both conventional and PCF-MS methods a series of peaks appeared at 337–339 *m*/*z*. These are doubly charged ions and, from their isotopic pattern, contain more than one Ru atom. These species are likely to be [(η^6^-indan)Ru_2_(μ-2,3-dpp) + (l-Cys)]^2+^, 338.49 *m*/*z* and [Ru_2_(μ-2,3-dpp) + 2(l-Cys)]^2+^, 339.99 *m*/*z*. However, the low intensity of the signals and overlap meant that these assignments were difficult to confirm. Two other photoproducts were also detected and are shown in Fig. 4B. These are [(η^6^-indan)Ru(μ-2,3-dpp) + (l-Cys)]^+^, 574.09 *m*/*z*, and [(η^6^-indan)Ru_2_(μ-2,3-dpp) + 2(l-Cys)]^2+^ at 396.52 *m*/*z*.

Reactions with the tripeptide glutathione (γ-l-Glu-l-Cys-Gly, GSH) were studied since this tripeptide often plays an important role in the cellular mechanism of resistance to metal-based drugs.^37^–^39^ Complex **1** (250 μM) was irradiated in the presence of two molar equiv. glutathione (GSH), see Fig. 4B and D, with the dark controls again incubated for the same length of time as the period of irradiation (30 min for the PCF-MS experiments and 14 h for the sample analysed conventionally). The dark control shows a new species at 408.56 *m*/*z* that contains one Ru atom, is doubly charged, and appears to contain one molecule of GSH and a fragment of GSH bound with the loss of an indan group. This species was present even in the dark but its intensity decreased after irradiation. In the spectrum of the irradiated reaction more new species were observed including that at 430.52 *m*/*z* assigned as [(η^6^-indan)Ru_2_(μ-2,3-dpp) + GSH]^2+^, and 526.06 *m*/*z* as [(η^6^-indan)Ru + GSH]^+^. Species at around 580 and 584 *m*/*z* form a cluster of peaks in the PCF-MS spectrum (Fig. 4D), but in the spectrum of the conventionally irradiated sample only the 584.06 *m*/*z* species, [(η^6^-indan)Ru_2_(μ-2,3-dpp) + 2GSH]^2+^ is present.

## Conclusions

Hollow-core photonic crystal fibre microreactors offer an integrated technique for the analysis of photochemical reactions. The combination of a low-volume microfluidic, continuous flow circuitry with high optical intensities in the HC-PCF renders the approach much less sample- and time-consuming than cuvette-based analytical approaches. This novel technique has been used here to gain new insight into reactions of potential importance to the biological mechanism of action of the dinuclear ruthenium(ii) complex **1**, [{(η^6^-indan)RuCl}_2_(μ-2,3-dpp)](PF_6_)_2_. Complex **1** was used for exploratory PCF-MS studies as it is a positively-charged photoactivatable metal complex with potential to form novel adducts with biomolecules and so introduce a new mechanism of metallodrug action. The compound gave a high MS signal intensity when flowed through the system and unambiguous assignment of photoproducts from the complex was readily achieved. The spectra from PCF-MS experiments and controls based on conventional irradiation methods showed no differences other than a small reduction in intensity of peaks for the PCF-MS spectra. All the detected species were the same by both methods. However, the time needed for sample irradiation and the total sample volume was dramatically reduced by using the PCF-MS method, from 14 hours to 12 seconds (the residence time in the HC-PCF core). Upon irradiation of **1**, by both methods, there was an increase in the species at 227.04 *m*/*z* assigned as [(η^6^-indan)Ru(2,3-dpp)]^2+^, suggesting that exposure to light can result in new reaction pathways, including loss of an arene ligand.

Having established the suitability of the PCF-MS system to study the irradiation of **1**, a series of experiments using a range of small molecules to act as models for larger species present within cells was carried out to gain insight into reactions that might be relevant to its biological activity. Guanine N7 is often a favoured binding site for metal-based drugs, and in the presence of the nucleotide guanosine 5′-monophosphate, 5′-GMP,^35^,^36^ **1** undergoes aquation and forms a mono-adduct. This is supported by the findings of Magennis *et al.* who reported DNA binding of **1** in the dark.^33^ When **1** was irradiated in the presence of 5′-GMP, new 5′-GMP adducts were detected. The binding of 5′-GMP that occurs following aquation of the complex also increases in the post-irradiation period.

Similarly, when photoactivated in the presence of 5′-AMP, adducts of 5′-AMP with **1** were also detected, but the peaks attributed to these are less intense than products formed by 5′-GMP. Magennis *et al.* also reported an increase in DNA binding upon irradiation.^33^

The reaction of **1** with l-Cys is consistent with the findings of Wang *et al.* who reported that [(η^6^-biphenyl)Ru (en)Cl]^+^ reacts slowly with l-cysteine in aqueous solution.^5^ Complex **1** also binds to the cysteine-containing tripeptide glutathione, which is present in cells at millimolar concentrations, as indicated by the detection of the peaks at 430.52 *m*/*z*, 526.06 *m*/*z* and 584.06 *m*/*z* assignable to [(η^6^-indan)Ru_2_(μ-2,3-dpp) + GSH]^2+^, [(η^6^-indan)Ru + GSH]^+^ and [(η^6^-indan)Ru_2_(μ-2,3-dpp) + 2GSH]^2+^ respectively. Binding to GSH following photoactivation of **1** may influence its biological activity. Hence these experiments suggest that this PCF-MS system will be useful as a high-throughput method of screening for light-activated drugs, providing insight into reactions of importance to drug design and activity.

Several improvements to our current system can be envisaged: firstly, the overall time for data acquisition could be minimized by reducing the internal dead volume of the HR-MS, for instance by electrospray ionisation directly from the optofluidic interface chip.^40^–^42^ This would allow the detection of (even) shorter-lived reaction species that are undetectable using conventional techniques. In addition, the current system could be combined with in-fibre spectroscopic methods.^25^ This would allow simultaneous excitation, optical detection and mass-spectrometry analysis of reaction products. Finally, we foresee that our approach can be combined with other lab-on-a-chip functionalities^40^,^43^ for sample preparation, separation and (multimodal) analysis in a straightforward fashion. In such an integrated system, laser light could be delivered by fibre-to-fibre coupling between the HC-PCF and embedded standard optical fibres, allowing easy operation without alignment of optical components.

Finally, we have used blue light in the current studies, but longer wavelength light penetrates more deeply into tissues. It would therefore be interesting to use our system to probe the metallodrugs that are optimised for excitation at longer wavelengths. An alternative strategy would be to employ a two-photon excitation process, using *e.g.* pulsed 800 nm wavelength light to achieve activation at 400 nm. Liquid-filled HC-PCFs could be used to study and optimise such nonlinear processes, as we recently demonstrated for two-photon excitation of fluorescein in a HC-PCF.^44^

## Conflict of interest

No conflicts of interest to declare.

## Supplementary Material

Supplementary information
