# Supplementary material for: Rapid screening of photoactivatable metallodrugs: photonic crystal fibre microflow reactor coupled to ESI mass spectrometry
Source: RSC Adv. 2017 Jul 26;7(59):37340–8. doi: 10.1039/c7ra06735f (PMC5735366; doi:10.1039/c7ra06735f)
Supplement: Supplementary file 1 [file RA-007-C7RA06735F-s001.pdf]

Electronic Supplementary Material (ESI) for RSC Advances.
This journal is © The Royal Society of Chemistry 2017

Supplementary Information

Table S1 Possible structures for fragments of **1** and the products of its photodecomposition, as detected by MS.

m/z	Formula	Structure
372.0	$[\{(\eta^6\text{-indane})RuCl\}_2(\mu\text{-2,3-dpp})]^{2+}$	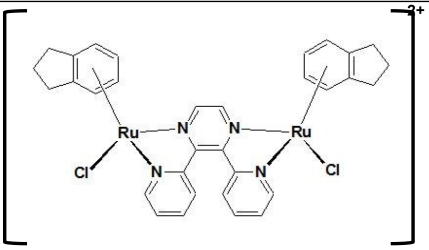
888.96	$[[\{(\eta^6\text{-indane})RuCl\}_2(\mu\text{-2,3-dpp})](PF_6)]^{+}$	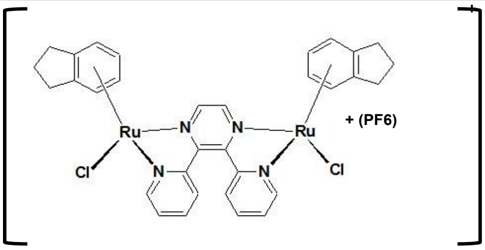
489.01	$[(\eta^6\text{-indane})RuCl(\mu\text{-2,3-dpp})]^{+}$	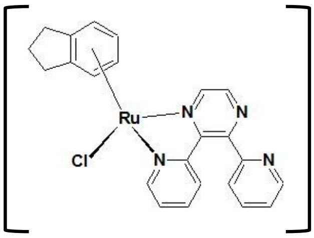
276.99	$[(\eta^6\text{-indan})Ru_2(\mu\text{-2,3-dpp})]^{2+}$	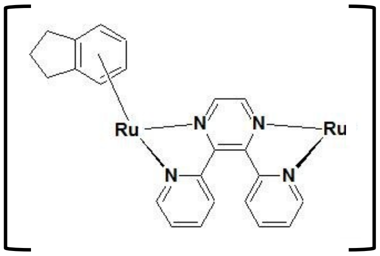
227.04	$[(\eta^6\text{-indan})Ru(\mu\text{-2,3-dpp})]^{2+}$	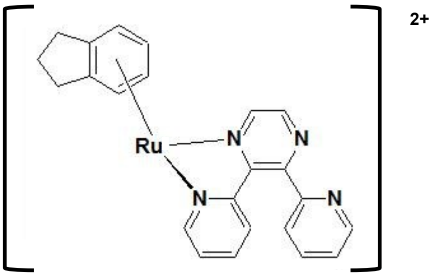
